# Exploring the Mechanism of Zanamivir Resistance in a Neuraminidase Mutant: A Molecular Dynamics Study

**DOI:** 10.1371/journal.pone.0044057

**Published:** 2012-09-06

**Authors:** Nanyu Han, Xuewei Liu, Yuguang Mu

**Affiliations:** 1 School of Biological Sciences, Nanyang Technological University, Singapore, Singapore; 2 School of Physical and Mathematical Sciences, Nanyang Technological University, Singapore, Singapore; Centers for Disease Control and Prevention, United States of America

## Abstract

It is critical to understand the molecular basis of the drug resistance of influenza viruses to efficiently treat this infectious disease. Recently, H1N1 strains of influenza A carrying a mutation of Q136K in neuraminidase were found. The new strain showed a strong Zanamivir neutralization effect. In this study, normal molecular dynamics simulations and metadynamics simulations were employed to explore the mechanism of Zanamivir resistance. The wild-type neuraminidase contained a 3_10_ helix before the 150 loop, and there was interaction between the 150 and 430 loops. However, the helix and the interaction between the two loops were disturbed in the mutant protein due to interaction between K136 and nearby residues. Hydrogen-bond network analysis showed weakened interaction between the Zanamivir drug and E276/D151 on account of the electrostatic interaction between K136 and D151. Metadynamics simulations showed that the free energy landscape was different in the mutant than in the wild-type neuraminidase. Conformation with the global minimum of free energy for the mutant protein was different from the wild-type conformation. While the drug fit completely into the active site of the wild-type neuraminidase, it did not match the active site of the mutant variant. This study indicates that the altered hydrogen-bond network and the deformation of the 150 loop are the key factors in development of Zanamivir resistance. Furthermore, the Q136K mutation has a variable effect on conformation of different N1 variants, with conformation of the 1918 N1 variant being more profoundly affected than that of the other N1 variants studied in this paper. This observation warrants further experimental investigation.

## Introduction

Since the beginning of last century, several pandemics caused by influenza A viruses have occurred, taking many lives [1]. These pandemics arise as new strains of influenza viruses emerge through re-assortment of the same or different subtypes during co-infection of different host species [2], [3]. The new virus strains are often highly lethal, because humans have not developed prior immunity [4], [5]. To date, 16 types of hemagglutinin (H1–H16) and 9 types of neuraminidase (N1–N9) have been reported [6].

Vaccines and antiviral drugs are two available strategies for preventing and controlling influenza virus infections. It takes three to six months to create a vaccine for a newly emerged virus strain. Such delay in provision of effective prophylactic measures may provide an opportunity for the global dissemination of the strain and may cause significant morbidity among human hosts worldwide [7]. The lag in vaccine manufacturing places limits on the effectiveness of vaccine applications, and medical experts are investigating the alternative of antiviral therapy for controlling novel infections [8]. Currently, several types of inhibitors are available to treat this disease, such as M2 inhibitors (amantadine and rimantadine) as well as neuraminidase (NA) inhibitors (Oseltamivir, Zanamivir (ZMR), and Peramivir) [7]. However, researchers have found numerous cases of drug-resistance to the M2 inhibitors. Due to the danger of drug-resistant mutations, the use of M2 inhibitors should be judiciously considered [9].

To date, the most effective drugs in use are the NA inhibitors. NA functions by cleaving away the sialic acid on host cells, to release nascent viruses that can then invade other host cells [10]. The NA inhibitors are designed to mimic sialic acid [11], and compete with sialic acid for the binding sites in NA. These inhibitors block the cleaving process, and thus impede viruses from spreading to other target cells. The drug Oseltamivir has high oral bioavailability, and can be formulated as capsules or powder for liquid suspension. Some countries stockpile this drug for contingency reserves [12]. By comparison, ZMR is a less favorable drug, as it can only be administered via inhalation [6].

Due to extensive use of Oseltamivir, influenza strains with NA mutations, such as H274Y, N294S, Y252H, I223R/V, and many others, have developed resistance to this drug [13], [14], [15], [16], [17]. Interestingly, nearly all the Oseltamivir-resistant strains are susceptible to ZMR [18]. One reason could be that the unique structure of ZMR nullifies the resistance developed to Oseltamivir. The molecular structure of ZMR includes a guanidine group, instead of the amino group found in Oseltamivir and in sialic acid [10]. In addition, while Oseltamivir has a hydrophobic group side chain, ZMR has a glycerol side chain. This hydrophilic side chain may prevent drug resistance, due to the hydrophobic pocket modification of mutant NA strains [14]. Furthermore, ZMR has had limited use to date, so the emergence of resistance to this drug is still not yet fully understood [18].

Recently, Hurt et al. reported influenza strains that carried a mutation on glutamine 136 (Q136K) in NA (H1N1) [19]. This mutant was resistant to ZMR, with a 300-fold reduction in susceptibility. However, these strains remained sensitive to Oseltamivir [19]. It is less common to find viral strains with ZMR neutralization effect than it is to find strains with resistance to Oseltamivir. It remains unclear why this is so. The Q136K strain's mutation position is distant from the drug binding sites, and it is not yet determined how the Q136K mutant develops ZMR resistance. The solution to this puzzle may provide valuable information for drug design.

**Figure 1:**
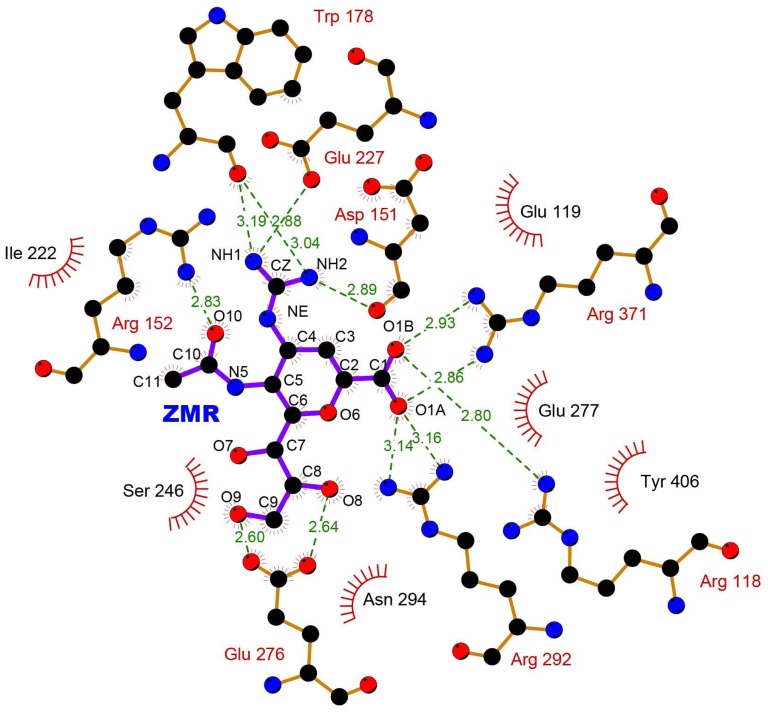
Interaction between active site residues and zanamivir plotted by ligplot from PDBsum website [**22**], [**24**]. ZMR is shown as ribbon with purple color, and active site residues are colored in brown. Carbon, nitrogen and oxygen atom is shown as black, blue and red balls. Green dashed lines represent hydrogen bond between ZMR and active site residues. Non-bonded contacts are shown as dark red “eyelashes”.

NA is a mushroom-shaped homotetrameric transmembrane glycoprotein that is localized in the outer layer of the virus envelope [20]. The head region of each sub-unit consists of six beta-sheets, forming a six-bladed propeller-like structure. Each sub-unit has a complete binding pocket, and binding sites are conserved among different subtypes [21]. Figure 1 (generated from PDBsum [22], [23] using ligPlot software [24]) illustrates the interaction between ZMR and the binding sites of this protein. NA has a pivotal structure motif, known as 150 loop, or 150 cavity (residues 147–152) [21], [25]. The various forms that this loop takes, such as open or closed, can exert a great influence on drug recognition and binding [17], [20], [21]. Some NA subtypes may change conformation, and thus alter the shape of the binding pocket during ligand binding process [21].

In this study, normal molecular dynamics (MD) and metadynamics simulations [26] were employed to explore the structural and dynamical properties of wild type (WT) and Q136K mutant NA. MD simulations of the apo forms of NA aimed to discern the conformational differences between WT and the mutant. Holo-form simulations helped in determining the structural differences of the ZMR binding mode between WT and Q136K mutants, and delineating the underlying molecular mechanism of resistance. Metadynamics simulations can help to overcome free-energy barriers [27]. Therefore, we employed these simulations to study the association/dissociation events and the free-energy landscape of the protein-drug complex in these systems.

## Methods

### Normal MD simulations

Normal MD simulations of both apo and holo forms of WT and Q136K neuraminidases were performed. Partial charges of ZMR were calculated by Gaussian09 [28], using the R.E.D.-III.4 tool [29]. Atom types were assigned by the antechamber program [30], which is included in the AMBER10 package [31]. The NA protein sequence was translated from the DNA sequence published by Hurt et al. [19]. A sequence-similarity search was performed using BLASTP [32]. An entry of 3B7E in PDB [33] (1918 A/H1N1) was identified as the most similar sequence, as this was 93% positive and 89% identical to the query NA sequence. Therefore, the structure of 1918 N1 (3B7E) was used as the WT holo structure. In addition, to check whether the mutant effect applied to the current H1N1 virus strain, the recently resolved structure, pdm09 H1N1 (3NSS [34]) was also used. These two proteins were first aligned, and then the holo structure of the pdm09 WT N1 was constructed by adding ZMR to the binding pocket of the apo form. For a highly lethal virus strain, H5N1 [35], available PDB crystal structures were also analyzed. However, no WT crystal structure bound with ZMR. One crystal structure (2HU4 [21]), which was co-crystallized with the drug Oseltamivir seemed worthy of further attention. After carefully comparing the crystal structure with that of the two other H1N1 viruses, it was found that some residues in the 150 loop and other binding sites were taking different backbone conformations. These differences in conformation influenced the binding pattern of ZMR so greatly that it was not valid to use this structure as the holo form.

The mutant was built by removing side chain atoms of Gln 136, changing the residue name to Lys, and then adding its side chain by tleap, using the Amber ff99SB force field [36]. For the apo form of the 1918 N1 strain (3B7E), Zanamivir was removed from the PDB file. The original crystal structure of the pdm09 N1 strain (3NSS) was used as its apo form. Other MD simulation procedures were the same as for the holo form.

Each of these systems were solvated with TIP3P water in a cubic box, where the minimal distance between atoms in complex (holo) or protein (apo) forms and the edges of the box was set to 1.2 nm. All systems were neutralized by adding counter ions. The simulations were conducted at room temperature (300 K) [37] and a constant pressure of 1 bar [38] in an isothermal-isobaric ensemble. The GROMACS program, suite version 4.0.5, [39] and the Amber99SB force field [36] were used in all the simulations. Protonation states of histidine residues were determined by Chimera software [40]. All the disulfide bonds were properly formed using the CYX notation in Amber. Both the holo and apo form simulations were first equilibrated for 5 ns by restraining all the heavy atoms of complex (holo) or protein (apo) forms. Both were then run for 30 ns normal MD simulations. For each system, three independent simulations were performed by assigning different initial velocities. Details of the system setups are given in Table 1.

**Table 1:** Detailed information of all the MD simulation systems.

System	Method	Structure	No. of atoms	No. of Water (TIP3P)	Time (ns)	Repeated time
1918N1 WT (holo)	Normal MD	wtN1+zanamivir	51537	15245	30	3
1918Q136K (holo)	Normal MD	Q136K+zanamivir	50956	15050	30	3
1918N1 WT (apo)	Normal MD	wtN1	51503	15248	30	3
1918Q136K (apo)	Normal MD	Q136K	50928	15055	30	3
1918N1 WT (holo)	Metadynamics	wtN1+zanamivir	51537	15245	100	2
1918Q136K (holo)	Metadynamics	Q136K+zanamivir	50956	15050	100	2
2009N1 WT (holo)	Normal MD	wtN1+zanamivir	53176	15771	30	3
2009Q136K (holo)	Normal MD	Q136K+zanamivir	53180	15771	30	3
2009N1 WT (apo)	Normal MD	wtN1	53502	15894	30	3
2009Q136K (apo)	Normal MD	Q136K	53512	15896	30	3
2009N1 WT (holo)	Metadynamics	wtN1+zanamivir	53176	15771	70	2
2009Q136K (holo)	Metadynamics	Q136K+zanamivir	53176	15771	50	2

### Metadynamics simulation

To study the free energy surfaces of the complexes, well-tempered metadynamics [41] was performed on the holo forms of WT and Q136K. Different from normal MD simulations, the metadynamics algorithm could accelerate rare event sampling and help the system to escape from free-energy minima through properly chosen collective variables (CV) [26]. In metadynamics simulations with added history-dependent Gaussian potentials, MD simulations are biased and the sampling of phase space increases [42], [43]. Moreover, it has been shown that well-tempered metadynamics can resolve the convergence problem regarding fluctuation of the free energy landscape in normal metadynamics simulations. Such metadynamics can also allow greater computational focus on the physically relevant regions of the conformational space [41], [44]. A number of studies in areas such as protein folding [25], [45] or the ligand-target recognizing process [46], [47] have already verified the efficiency and accuracy of this algorithm.

The first collective variable (CV1) is the distance between the center of mass of the heavy atoms of ZMR, and the center of mass of the carbon α atoms of Phe121, Glu276 and Tyr406. These residues are located at the bottom of the active site. Here, the CV1 was used to monitor the closeness of ZMR to the binding site. CV2 is the radius of gyration of heavy atoms of residues located on the outer ring of the binding pocket. The residues included are 149, 150, 151, 152, 222, 223, 325, 326, 366, 367, 430, 431, 432, 433, 434, 435 and 436. Most of these residues are components of the 150 and the 430 loops. The 430 loop consists of residues from 429 to 433.

Several sets of parameters were assessed to check the convergence and accuracy [42], [43], [48] of the free energy profile. The final parameter set was chosen as ω = 0.3 kJ/mol (∼0.07 kcal/mol), τG = 1000. The deposition rate for the Gaussian bias functions was set to 2 ps in both WT and Q136K. The bias factor was 10 in this well-tempered metadynamics simulation. Other simulation parameters and conditions were the same as those in normal MD simulations.

The initial structure was obtained from the final snapshot of the normal MD simulation. The metadynamics simulations ran for 100 ns for both the WT and Q136K of 1918 N1 systems. For the 2009 N1 system, Q136K was stopped at 50 ns, and WT simulated for 70 ns. These simulations were performed using the Gromacs package, augmented with a metadynamics package known as Plumed [49]. The metadynamics simulations were repeated two times for each system, using different initial velocities.

## Results and Discussion

### The 3_10_ helix before the150 loop is absent in the apo form of Q136K mutant

**Figure 2:**
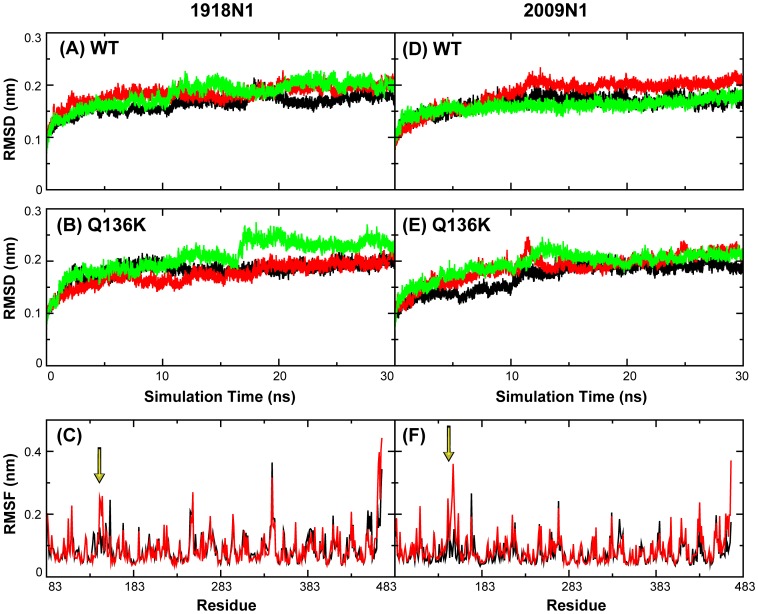
Root mean square deviation (RMSD) and fluctuation (RMSF) in two systems of two H1N1 viruses. Root mean square deviation (RMSD) of heavy atoms of WT (Figure 2A) and Q136K (Figure 2B) in 1918 N1. Figure 2D and 2E show heavy atoms RMSD of WT and Q136K of 2009 N1. Black, red, green color lines represent three times simulation of apo form structures. Figure 2C and 2F show Root mean square fluctuation (RMSF) of each residue during apo form simulations for 1918 N1 and 2009 N1. Black and red lines represent WT and Q136K respectively.

Both the WT and Q136K NAs of two H1N1 strains were stable during the 30 ns simulations, with the overall root-mean-squared deviations with respect to the crystal structure less than 0.25 nm (see Figure 2). The root-mean-squared fluctuations (RMSF) for each residue are also shown, in Figures 2C and 2F. Comparing residues from 143 to 147, the Q136K displayed larger fluctuations than the WT in both of the two N1 strains. This phenomenon was observed in all the apo-form simulations. The average RMSF values of these residues are listed in Table 2. For each system, three trajectories with different initial velocities were obtained. All of these showed consistent differences between the WT and the mutant indicating that the observed differences are statistically meaningful.

**Table 2:** Average of root mean square fluctuation (RMSF) value during 3 times apo form simulation.

Rmsf (nm)	WT (1918N1)	Q136K (1918N1)	WT (2009N1)	Q136K (2009N1)
K143	0.21 (0.01)	0.25 (0.03)	0.21 (0.01)	0.24 (0.02)
H144	0.16 (0.03)	0.21 (0.05)	0.14 (0.01)	0.20 (0.01)
S145	0.08 (0.01)	0.15 (0.05)	0.07 (0.01)	0.20 (0.07)
N146	0.13 (0.01)	0.20 (0.04)	0.13 (0.01)	0.32 (0.10)
G147	0.11 (0.01)	0.17 (0.04)	0.13 (0.03)	0.25 (0.02)

Value in the parentheses is standard deviation.

**Figure 3:**
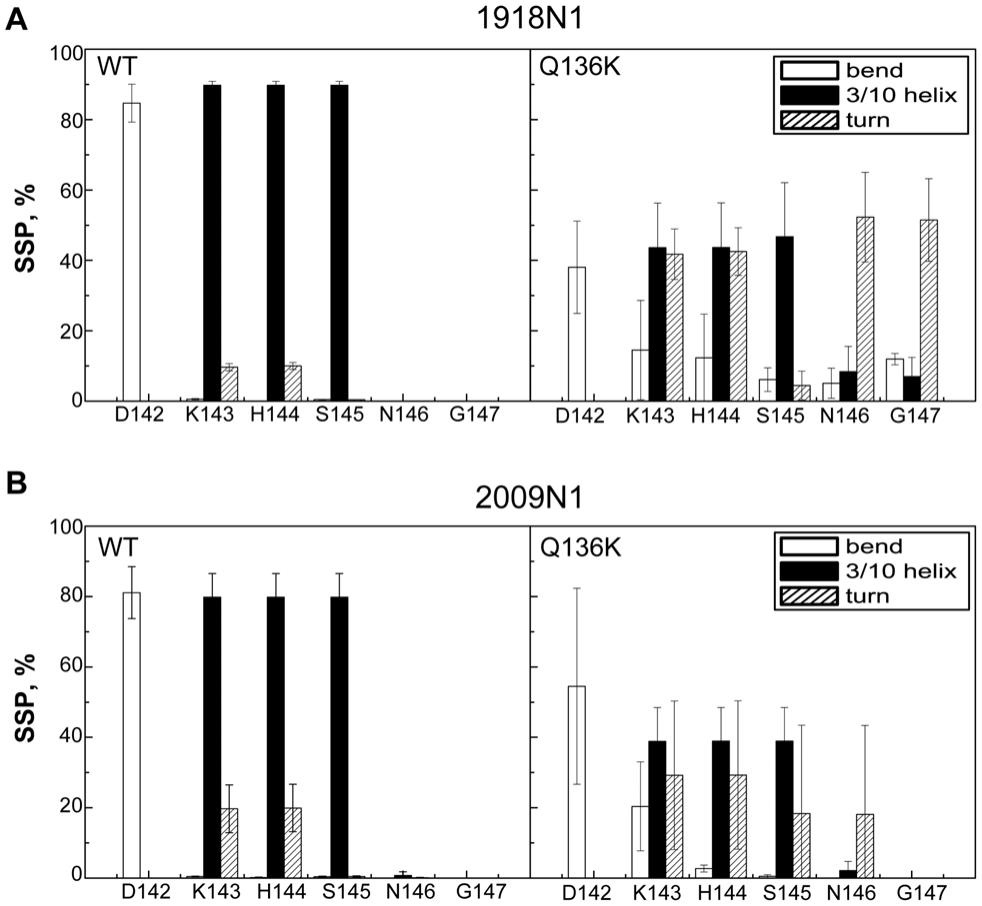
Secondary structure propensity of residues in two systems of two H1N1 viruses. Secondary structure propensity of residues in WT and Q136K of 1918 N1 (Figure 3A) and 2009 N1 (Figure 3B) respectively. The secondary structures were assessed by DSSP package.

To investigate what happened with these residues during the simulations, the secondary structure propensity was analyzed (Figure 3A, 3B). The residues from 143 to 145 had a 90% propensity to form 3_10_ helices in WT of 1918 N1, and an 80% propensity in 2009 N1. However, this propensity decreased to no more than 50% in the Q136K of both strains. The time evolution of the secondary structure demonstrated the process of conformational conversion (Figure S1). The 3_10_ helix structure stayed intact during the whole trajectory of the WT. However, in 1918 Q136K, the residues before the 150 loop converted to random coil after 17 ns in the simulation. In 2009 Q136K, the helix structure also changed its conformation after 12 ns. Similar results were observed in the other two apo-form simulations.

### Local structures and dynamics are perturbed by K136

**Figure 4:**
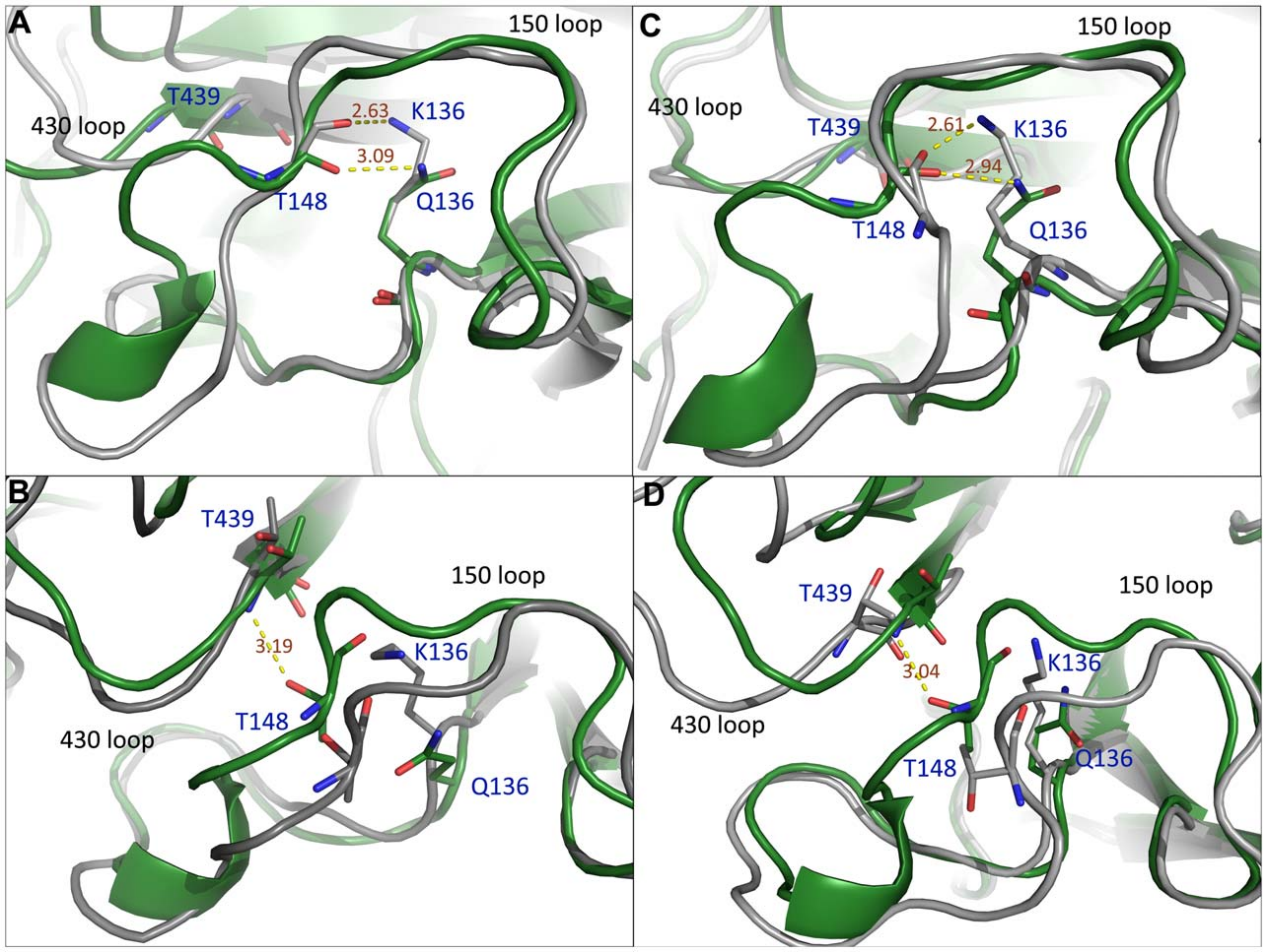
Structure comparison between WT and Q136K in two H1N1 viruses. Snapshot of WT (green) and Q136K (grey) of NA at t = 30 ns (Figure 4A for 1918 N1, 4C for 2009 N1). Representative structures of WT (green) and Q136K (grey) show the interaction between the 150 loop and the 430 loop (Figure 4B for 1918 N1, 4D for 2009 N1). The representative structure is the center of the largest cluster obtained in the whole simulation. Structures were superimposed by Pymol.

The mutation site 136 is not a component of the active site residues in NA, but it sits close to the 150 loop. In the 1918 N1 crystal structure, the distances between the nitrogen atom of the Q136 side chain and the carbonyl oxygen atoms of T148 and K150 are 2.91 Å and 3.15 Å respectively. In the 2009 N1 crystal structure, these values are 3.23 Å and 3.13 Å (Figure S2). Hydrogen bonds can form between these two pairs of atoms. In the Q136K mutant, a hydrogen bond was maintained between T148 and the side chain of K136 (Figure S3). However, due to the longer side chain of lysine, the interaction between T148 and K136 pushed T148 and the 150 loop away from their original positions. Snapshots of the superimposition of the WT and Q136K structures at 30 ns are shown in Figures 4A and 4C, for 1918 N1 and 2009 N1 respectively. The positions of T148 and residues before the 150 loop, plus the shape of the 150 loop, were both changed due to the mutated lysine residue. The T148 in Q136K moved forward as compared with its position in WT. Also, the helical secondary structure was absent in the mutant strain.

**Figure 5:**
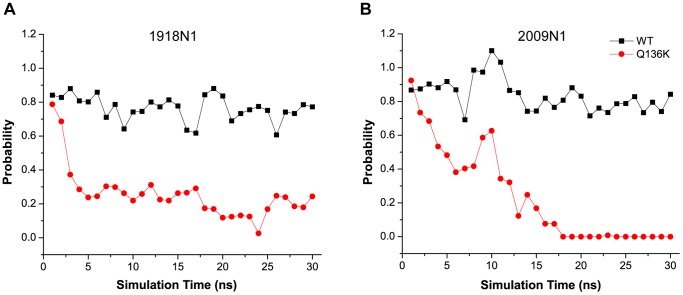
Probability of hydrogen bond formation between T148 and T439. Probability of hydrogen bond formation between T148 and T439 in WT (black) and Q136K (red) of time evolution in 1918 N1 (Figure 5A) and 2009 N1 (Figure 5B). The probability was analyzed every 1 ns during simulation, and the dots here represent average value of the 3 trajectories.

The time evolution of the backbone dihedral angles of G147 in Q136K showed different behavior from that of WT in both H1N1 strains. The phi angle of G147 mainly fluctuated around 90 degree in WT, but in Q136K this angle jumped to another cluster of angle around −90 degree (See Figure S4). In WT, there was a stable interaction between T148 and the 430 loop in both N1 strains (Figures 4B and 4D). The probability of a hydrogen bond forming between T148 and T439 was greater than 80% in WT. However, this probability decreased to 20% in Q136K after 5 ns simulation in 1918 N1, and this probability almost disappeared after 17 ns in the 2009 N1 simulation (Figure 5). Both the 150 loop and the 430 loop are components of the binding pocket, and thus are important for drug recognition and binding [50]. In the current study, the 150 loop in the Q136K mutant deviated from its original position. This perturbation of the 150 loop in the Q136K mutant may affect ZMR binding affinity.

### The interaction between ZMR and the active site residues in Q136K is weakened in the holo-form simulations

**Figure 6:**
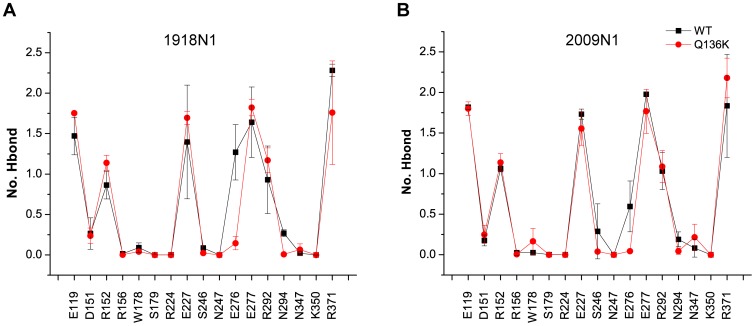
Hydrogen bond formation between ZMR and binding sites. Number of hydrogen bond formed between ZMR and active site residues in 1918 N1 (Figure 6A) and 2009 N1 (Figure 6B). Black and red lines represent WT and Q136K respectively.

The hydrogen bonds formed between ZMR and the active site residues were monitored. Most of the hydrogen bonds were maintained in the Q136K as in the WT (Figure 6). However, an evident difference appeared in the number of hydrogen bonds formed between E276 and ZMR. In WT, there was more than one hydrogen bond in the 1918 N1 strain. For 2009 N1, this hydrogen bond was also fully maintained in WT. However, this interaction was rarely maintained in the Q136K mutants of either H1N1 strain.

**Figure 7:**
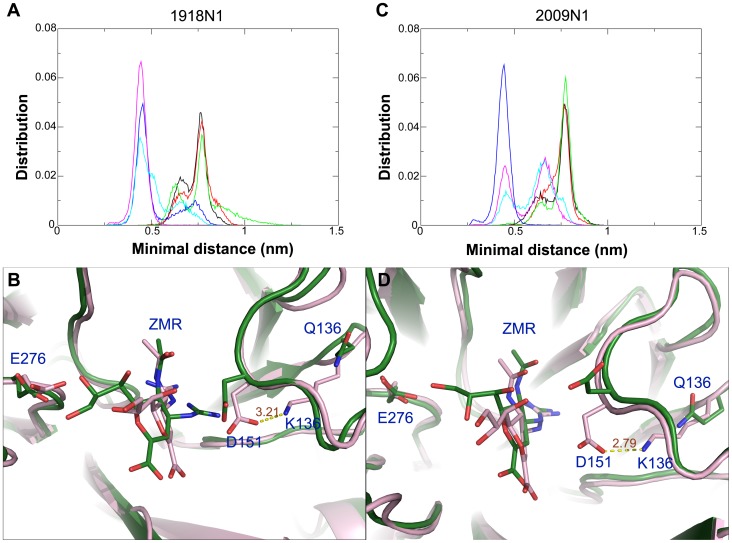
Distance distribution and conformation comparison between WT and Q136K. Minimal distance distribution between side chain of D151 and Q/K136. Lines in black, red and green color represent the distance distribution of the separate holo WT simulations, and lines in blue, magenta, cyan color in Figure 7A and 7C indicate Q136K simulations. The snapshot of WT and Q136K of holo form simulations at t = 30 ns, showing the interaction between ZMR and the active site residues. Structures in green and pink color indicate WT and Q136K in 1918 N1 (Figure 7B) and 2009 N1 (Figure 7D) respectively. Structures were superimposed by Pymol.

The loss of the hydrogen bond between ZMR and E276 was accompanied by a stronger interaction between D151 and K136. Distance distributions between D151 and K136 in the WT and Q136K are shown in Figures 7A and 7C. The minimal distance between side chains of D151 and Q136 was mainly distributed around 0.8 nm in WT, but this distance was reduced to around 0.4 nm in Q136K. This finding indicates that a strong salt bridge between K136 and D151 is present in the mutant. In the apo form of Q136K simulations, a similar salt bridge was also observed (Figure S5). A snapshot of ZMR tucked inside the active site, as well as the interaction between ZMR and active site residues, is shown in Figures 7B and 7D. In the WT NA, ZMR maintained close contact with E276 and D151 in both strains. In the Q136K mutant, however, the ZMR underwent a positional shift and lost contact with both E276 and D151. Originally, ZMR was designed by replacing the amino group of the sialic acid with a guanidine group, in order to gain favorable interaction with several residues, including D151 of the 150 loop. The loss of this beneficial interaction between ZMR and the active site residues in Q136K may be the cause for weakened inhibition effects.

In the reportedly Oseltamivir-resistant mutants such as H274Y and N294S [14], the mutated residues perturbed the orientation of the carboxyl group of E276. These mutants also altered the local hydrophobic environment, so that the pentyloxy group in Oseltamivir was no longer correctly positioned. The binding of ZMR and the native substrate (SIA) was less perturbed in these mutants, because they possessed a hydrophilic glycerol group instead of a pentyloxy group. Clearly, different drugs take dissimilar binding modes in the NA binding pocket. In the current study, the stability of the 150 loop was pivotal to the binding affinity of ZMR. Thus, the dynamics and conformation of the 150 loop should be taken into account in the design of new drugs.

### Metadynamics simulations

**Figure 8:**
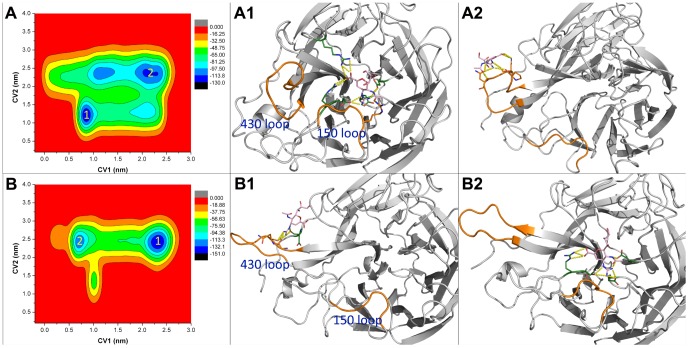
Free energy landscape and representing conformation of WT and Q136K in 1918 N1system. Free energy landscape of WT (Figure 8A) and Q136K (Figure 8B), here CV1 is the distance between ZMR and 3 carbon α atoms of residues located at the bottom of binding pocket. CV2 represents the radius of gyration of selected group of heavy atoms located at the outer ring of binding pocket. A1 and A2 show the representative structures of the 1 and 2 local minimum of Figure 8A, and B1 and B2 show the representative structures of the 1 and 2 local minimum of Figure 8B. The 150 loop and the 430 loop is shown in orange color, amino acids that interact with ZMR is shown in green color. Free energy landscape was plot with OriginPro 8, and structure figure was plot using Pymol.

The normal MD simulations revealed the local structural perturbations induced by the mutated residue. The free energy available for the binding or unbinding of drugs is the most relevant factor relating to drug resistance. Normal MD simulations have limitations in determining mutation effects in the free energy landscape. Therefore a well-tempered metadynamics simulation is one prospective means for interrogating the binding/unbinding process of the drug. In this study, the first CV (CV1) was chosen to monitor the binding/unbinding events. The normal MD simulations and other studies have shown that the flexibility of the 150 loop may lead to open and closed states of the binding pocket. Thus, the second CV (CV2) was used to sample the open and closed states of the binding pocket. The convergence of the metadynamics simulations was checked by examining the potential of the mean force of each CV (shown in Figure S6). The free energy surfaces of 1918 N1 and its mutant are shown in Figure 8. The free energy surface of the WT complex is different from that of the Q136K complex in 1918 N1. The conformation of drug-protein complex corresponding to the center of each local minimum was analyzed with the clustering method. In WT 1918 N1, the binding mode of the largest cluster (Figure 8A1) was similar to the representative structure of the normal MD simulations, with an RMS value of 0.082 nm. The typical structure of the normal simulation was also obtained from the clustering method, by choosing the center structure of the largest cluster. The representative structure, with large values of both CV1 and CV2, showed that ZMR was trapped near the 430 loop, and the configurations of both the 150 and the 430 loops did not change significantly in comparison to these loops in the crystal structure (Figure 8A2).

However, in the Q136K complex system, the representative structures of both dominant local minima exhibited a largely open binding pocket (Figures 8B1 and 8B2). The structure representing the lowest minimum showed that ZMR was trapped near the 430 loop, far away from the native binding site (Figure 8B1). The second-lowest local minimum had a free energy level much higher than the lowest one. The representative configuration in this case showed that ZMR was close to the native binding sites (Figure 8B2). The large value of CV2 in this configuration indicated an open conformation of the loops. These data verify the results obtained from the normal simulations, namely that the 150 and 430 loops of Q136K were more prone to deformation than those of WT.

**Figure 9:**
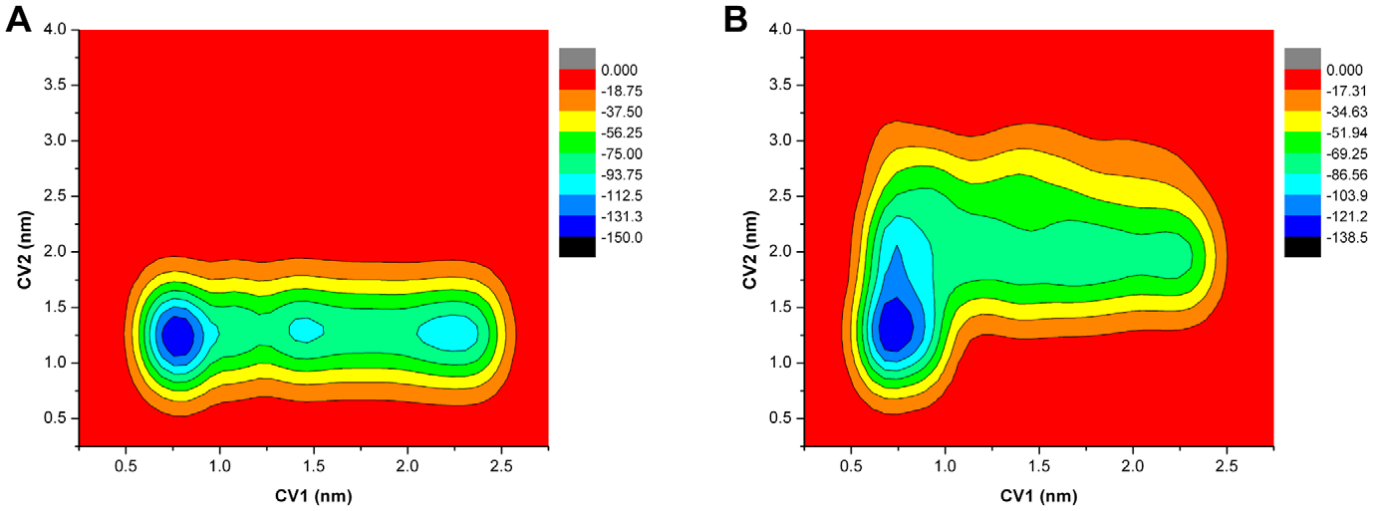
Free energy landscape of WT and Q136K in 2009 N1 system. Free energy landscape of WT (Figure 9A) and Q136K (Figure 9B), CV1 is the distance between ZMR and 3 carbon α atoms of residues locate at the bottom of binding pocket. CV2 represents the radius of gyration of selected group of heavy atoms located at the outer ring of binding pocket.

In metadynamics simulations of 2009 N1, the free energy surfaces of both WT and Q136K mutant possessed a deep local minimum, with small values of CV1. This indicated that the native binding pose was favorable, and ZMR was able to bind tightly with both WT and Q136K of 2009 N1 (Figure 9). In WT, there was no opening event for the loops until the metadynamics simulation reached 70 ns. In the Q136K mutant, however, the 150 and 430 loops were prone to conformational changes. Thus, similar to the situation with 1918 N1, the open configurations of the binding pocket for the 2009 N1 Q136K mutant were characterized by larger CV2 values and had lower free energy than configurations in the wild type. The crystal structure of 2009 N1 (3NSS) possessed a closed 150 loop that was different from other group 1 apo neuraminidase structures. This could be one reason why the 150 loop of 2009 N1 WT had a high probability of being closed, as demonstrated in the metadynamics simulations.

## Conclusion

In this study, normal MD simulations and metadynamics simulations were performed to explore the drug resistance mechanisms of the NA Q136K mutant. The mutated residue had significant perturbations affecting the stability of drug binding. Such perturbation occurred through conformational modification of the 150 loop. In the normal simulations of the apo and holo Q136K in both N1 systems, the local configurations were affected. The 3_10_ helix before the 150 loop vanished; the position of T148 on the 150 loop shifted due to interaction formed between T148 and the mutation site K136; the hydrogen bond linking the 150 loop with the 430 loop and the contacts between ZMR and E276/D151 disappeared.

The configuration of the 150 loop in Q136K did not show dramatic changes during the normal simulations. In metadynamics simulations, however, the 150 loop in the mutants took a conformation distinct from that of the wild type. Because the 150 loop is directly involved in the drug binding, it was not surprising that the free energy surface of the Q136K mutants was different from that of the wild type. On the perturbed free energy surfaces of mutants, the native binding mode was no longer the global minimum. Taken together, these findings indicate that the cause of Q136K ZMR drug resistance can be ascribed to the fact that the mutated K136 deforms the 150 loop, which then weakens the interaction between ZMR and the active site residues. Such a drug-resistance mechanism applies in the case of 1918 N1. However, in the case of 2009 N1, the perturbation effects of the Q136K mutation turned out to be weaker. Such variant–dependent phenomenon warrants further experimental investigations.

Our present study provides an atomic understanding of drug resistance in Q136K N1. Interestingly, such resistance is limited to Zanamivir only. The mutant is still sensitive to Oseltamivir. Other mutants such as H274Y and N294S, however, show resistance only to Oseltamivir. The difference is likely due to the distinct chemical structures of the drug molecules, the specific interactions between these drug molecules and the active site residues, and the different configurations of the 150 loop in the neuraminidase.

## Supporting Information

Figure S1 **Time evolution of the secondary structure of residues range from 138 to 146 for WT (A) and Q136K (B) of 1918 N1 system, and WT (C) Q136K (D) in 2009 N1 system.** The secondary structures were assessed by DSSP package.
(TIF)

Figure S2 **Interaction and distance between Q136K and 150 loop in crystal structure of 1918 N1 (A) and 2009 N1 (B).**
(TIF)

Figure S3 **Distance between K136 (NZ) and T148 (O) with the time evolution in 1918N1 and 2009 N1.**
(TIF)

Figure S4 **Ramachandran plot of G147 time evolution.** The first and last 3 columns show the backbone dihedral angle of WT and Q136K during 3 times simulation respectively. Figures in the first two, middle two and last two rows represent change of backbone dihedral angles in 1918 N1, 2009 N1 and H5N1 system respectively.
(TIF)

Figure S5 **Distribution of minimal distance between side chain of D151 and Q/K136 in apo form simulation in 1918 N1 and 2009 N1 system respectively.** Black, red and green lines represent distance distribution of three times WT apo simulation, blue, cyan and magenta color lines indicate distribution of Q136K during three times apo form simulations.
(TIF)

Figure S6 **Potential of Mean force for each collective variable at 90 ns (black) at 100 ns (red) in WT and Q136K 1918 N1 system respectively.**
(TIF)
