# Supplementary figures and images for: Exploring the Mechanism of Zanamivir Resistance in a Neuraminidase Mutant: A Molecular Dynamics Study
Source: PLoS One. 2012 Sep 6;7(9):e44057. doi: 10.1371/journal.pone.0044057 (PMC3435372; doi:10.1371/journal.pone.0044057)

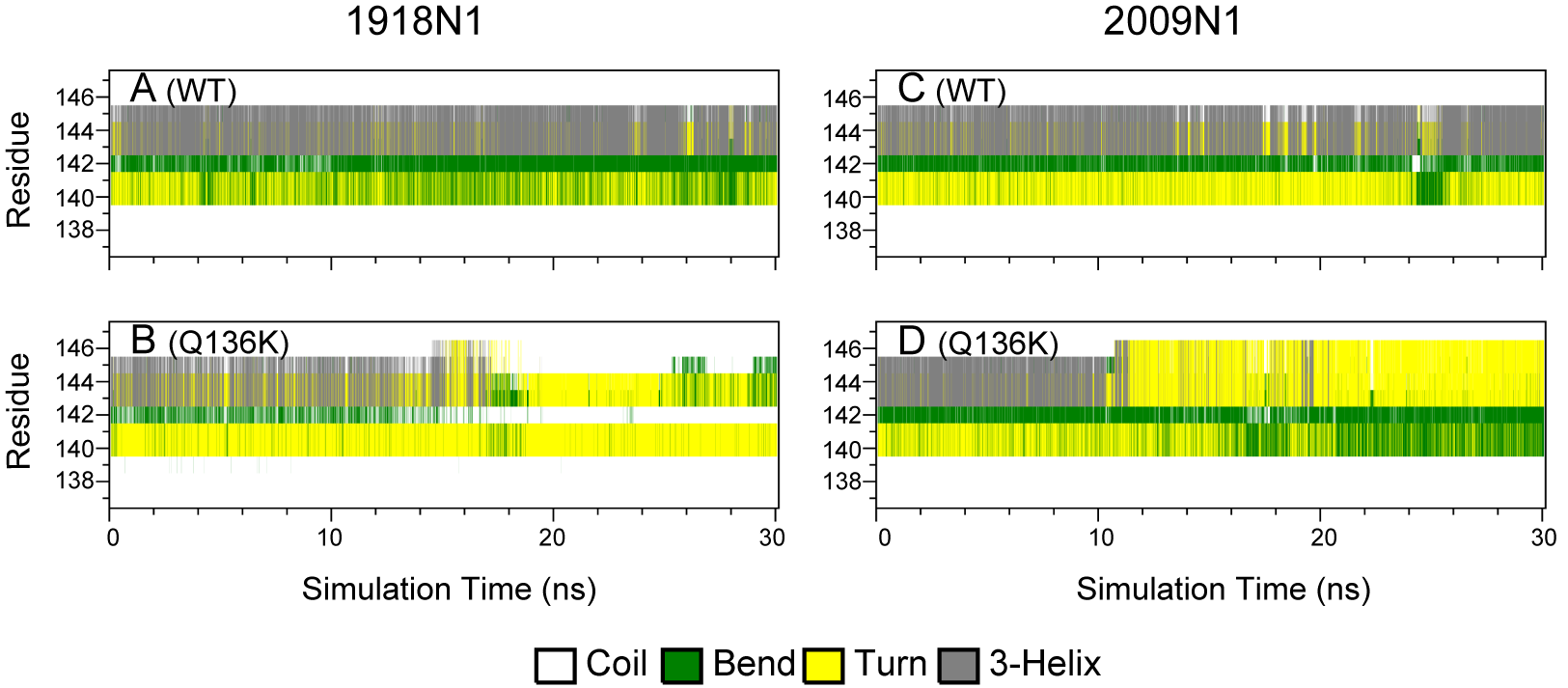

Supplement: Figure S1 — Time evolution of the secondary structure of residues range from 138 to 146 for WT (A) and Q136K (B) of 1918 N1 system, and WT (C) Q136K (D) in 2009 N1 system. The secondary structures were assessed by DSSP package. (TIF) [file pone.0044057.s001.tif]

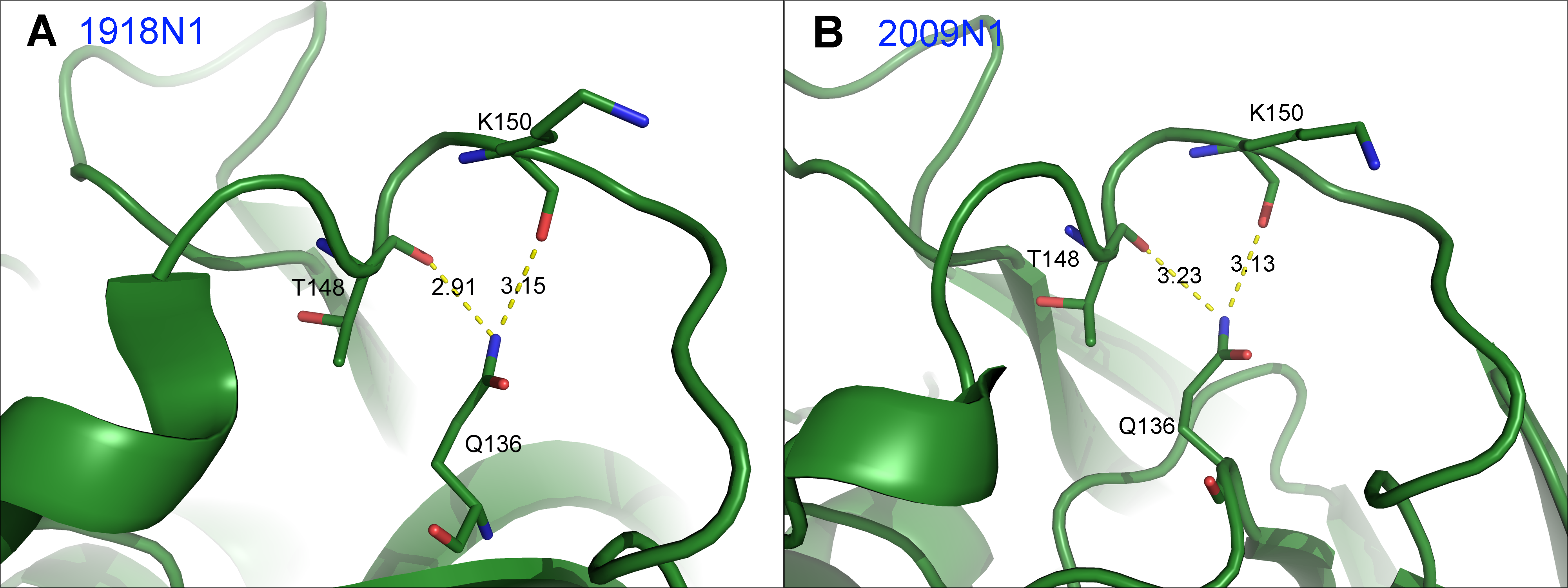

Supplement: Figure S2 — Interaction and distance between Q136K and 150 loop in crystal structure of 1918 N1 (A) and 2009 N1 (B). (TIF) [file pone.0044057.s002.tif]

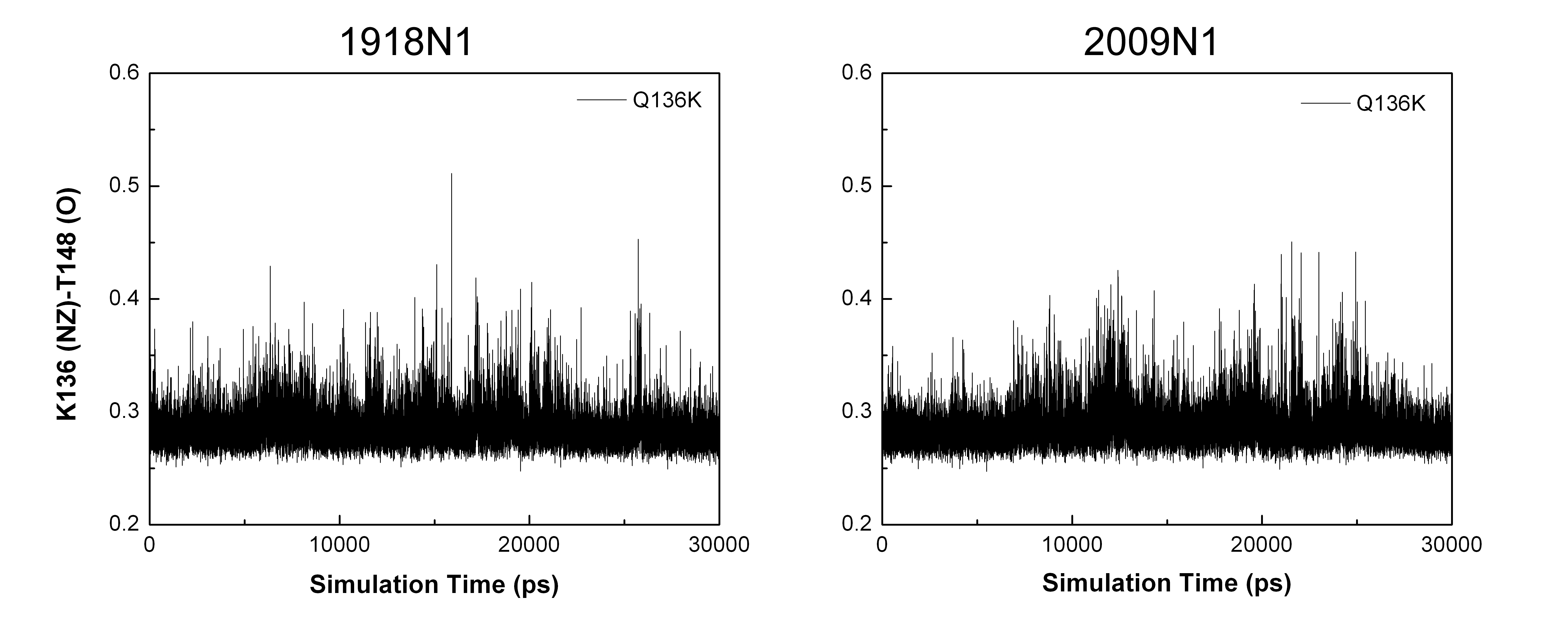

Supplement: Figure S3 — Distance between K136 (NZ) and T148 (O) with the time evolution in 1918N1 and 2009 N1. (TIF) [file pone.0044057.s003.tif]

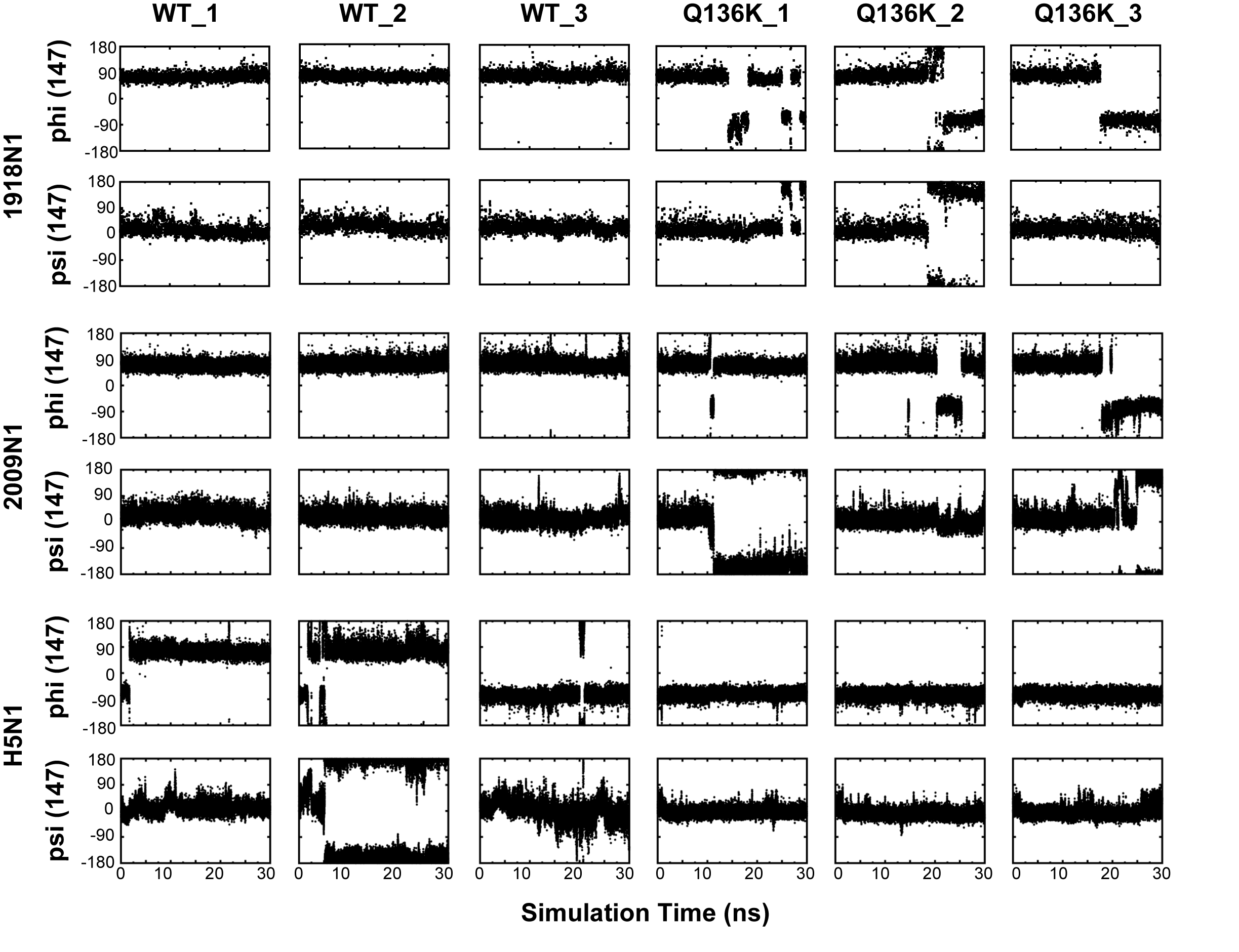

Supplement: Figure S4 — Ramachandran plot of G147 time evolution. The first and last 3 columns show the backbone dihedral angle of WT and Q136K during 3 times simulation respectively. Figures in the first two, middle two and last two rows represent change of backbone dihedral angles in 1918 N1, 2009 N1 and H5N1 system respectively. (TIF) [file pone.0044057.s004.tif]

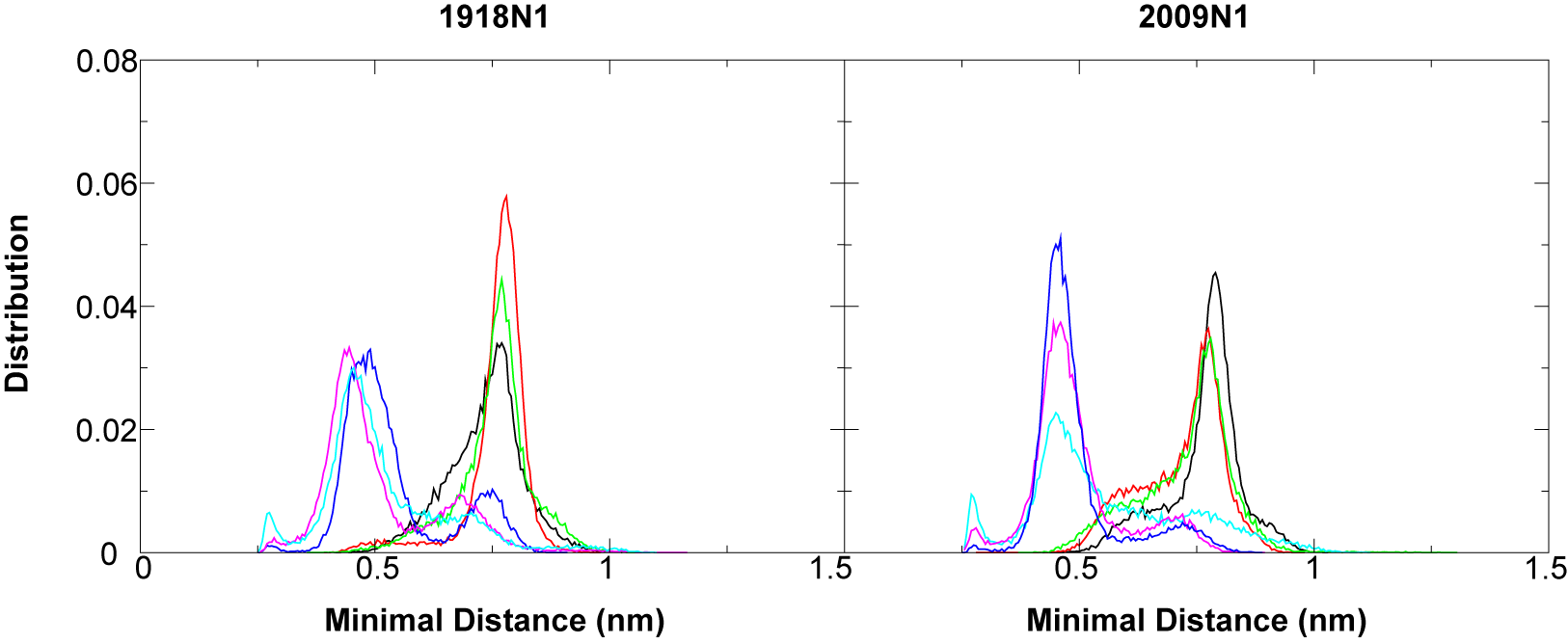

Supplement: Figure S5 — Distribution of minimal distance between side chain of D151 and Q/K136 in apo form simulation in 1918 N1 and 2009 N1 system respectively. Black, red and green lines represent distance distribution of three times WT apo simulation, blue, cyan and magenta color lines indicate distribution of Q136K during three times apo form simulations. (TIF) [file pone.0044057.s005.tif]

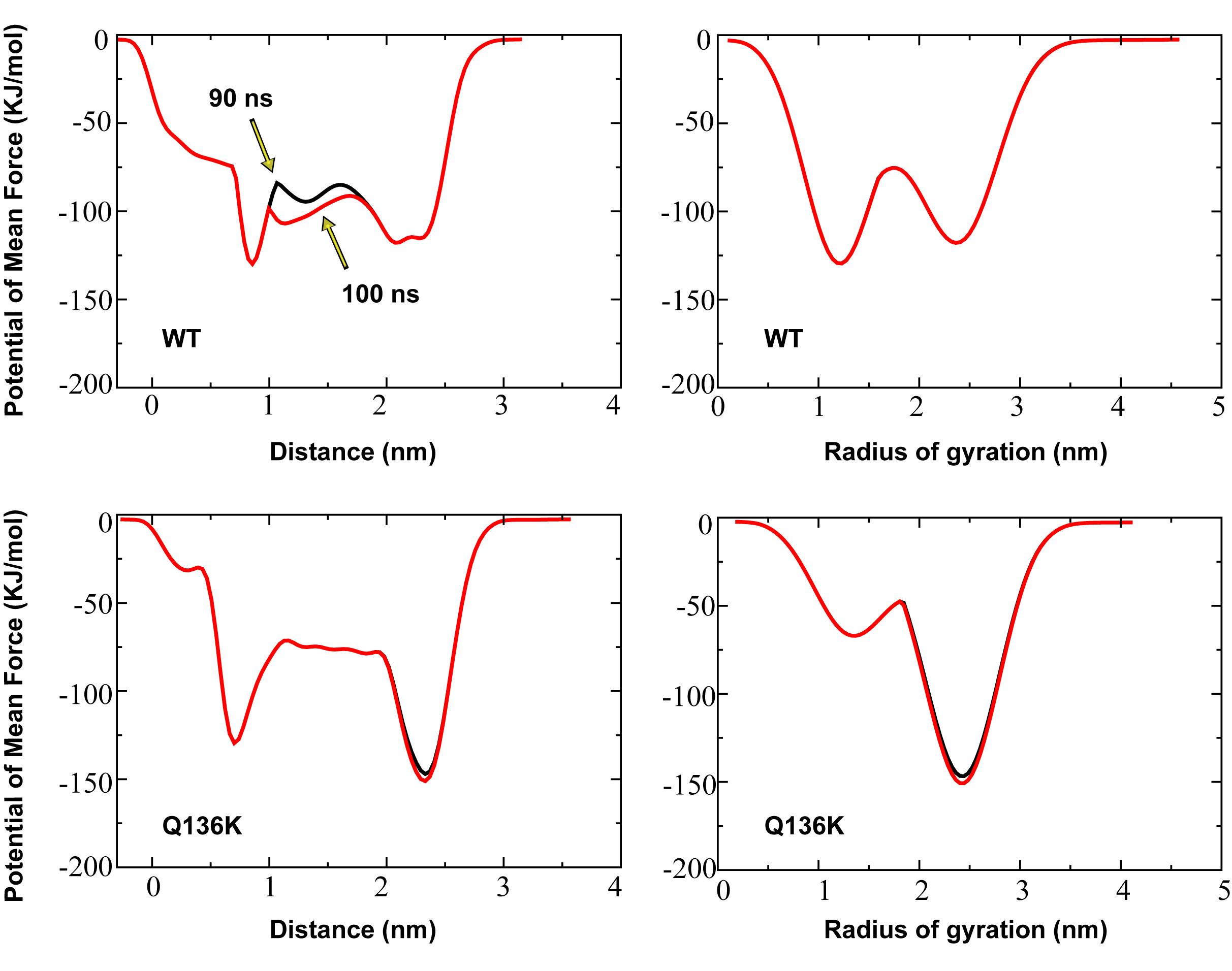

Supplement: Figure S6 — Potential of Mean force for each collective variable at 90 ns (black) at 100 ns (red) in WT and Q136K 1918 N1 system respectively. (TIF) [file pone.0044057.s006.tif]
